# CXCL10 Is Inhibited by PPARβ/δ-Selective Activation in Human Monocyte-Derived Dendritic Cells, T Lymphocytes and Monocytes In Vitro: Insights into Autoimmune Rheumatic Diseases

**DOI:** 10.3390/biom16091325

**Published:** 2026-09-11

**Authors:** Gabriele Farina, Mariangela Sottili, Giada La Spina, Stefania Gelmini, Simona Truglia, Fabrizio Conti, Francesca Romana Spinelli, Clara Crescioli

**Affiliations:** 1 Department of Movement, Human and Health Sciences, University of Rome Foro Italico, 00135 Rome, Italy; g.farina2@studenti.uniroma4.it; 2 Department of Experimental and Clinical Biomedical Sciences “Mario Serio”, University of Florence, 50121 Florence, Italy; mariangela.sottili@gmail.com (M.S.); bettiblu2002@gmail.com (S.G.); 3 Department of Internal Medicine and Medical Specialties, AOU Policlinico Umberto I, 00185 Rome, Italy; giada.laspina@uniroma1.it (G.L.S.); dott.trugliasimona@gmail.com (S.T.); 4 Rheumatology Unit, Department of Clinical Internal, Anaesthesiological and Cardiovascular Sciences, Sapienza University of Rome, 00185 Rome, Italy; fabrizio.conti@uniroma1.it (F.C.); francescaromana.spinelli@uniroma1.it (F.R.S.)

**Keywords:** CXCL10, PPARβ/δ, ARD, human immunocytes

## Abstract

The chemokine CXCL10 contributes to early autoimmune rheumatic disease (ARD) development. Preclinical ARD stages exhibit dendritic cells (DCs), CD4^+^ T cells, and monocytes (CD14^+^ cells) dysregulation and an overreactive immunoprofile. The peroxisome proliferator-activated receptor (PPAR)β/δ acts as a “molecular brake”, limiting autoimmune overresponse. This study aims to investigate CXCL10 in human monocyte-derived mDCs, CD4^+^ T cells, and monocytes exposed to PPARβ/δ agonist carbaprostacylin (cPGI_2_) or PPARγ agonist rosiglitazone (RGZ), to verify drug-class-related effects. Tumor necrosis factor (TNF)α, interferon (IFN)γ, synergistic inducers of CXCL10, and interleukin (IL)-10 were investigated. mDCs, CD4^+^ T cells, and CD14^+^ monocytes isolated from healthy donors’ blood were cultured, specifically activated to mimic an overreactive phenotype, and treated for 24–48 h with/without [10 μM] cPGI_2_ or [5 μM] RGZ (near therapy doses, respectively). Luminex assays quantified cell supernatant analytes; Real-time q-PCR measured cytokine and PPARβ/δ mRNA expression. cPGI_2_, not RGZ, significantly decreased CXCL10 in mDCs and CD4^+^ T cells, reduced TNFα in mDCs, CD4^+^ T and monocytes, and diminished IFNγ in mDCs. IL-10 did not change. cPGI_2_-induced CXCL10 downregulation, with TNFα and IFNγ decrease, might be relevant, given its early activity in ARD development. Scenarios on PPARβ/δ agonist re-positioning aimed at modulating CXCL10 might open opportunities to support ARD management and targeted therapy development.

## 1. Introduction

Autoimmune rheumatic diseases (ARD) are complex disorders, characterized by an excessive self-reactive immune response, severe clinical manifestations, and poor quality of life [1]. In ARD pathogenesis, determinant factors include genetic predisposition; infections; environmental insults, i.e., from chemical or physical agents; hormone status alteration; and stressful life events [1,2,3,4,5].

An aberrant cascade of cytokines and chemokines is fully recognized as a decisive etiopathogenetic issue in the main ARD subgroups—autoinflammatory, autoimmune, and overlapping diseases [1,6,7].

In particular, a family of small (8–10 kDa) highly chemotactic cytokines, named chemokines, can control immunocyte migratory pattern and positioning, with important pathogenic and therapeutic implications [8,9].

Patients affected by different types of ARD, like systemic lupus erythematosus (SLE), systemic sclerosis (SSc), rheumatoid arthritis (RA), vasculitis, or idiopathic inflammatory myopathies (IIM), show increased levels of chemokines and their receptors both in blood and tissues [9].

The chemokine interferon gamma (IFNγ)-inducible protein 10 (IP-10), also known as C-X-C motif chemokine ligand 10 (CXCL10), is a glutamic acid–leucine–arginine motif negative (ERL-) molecule that, upon binding its specific receptor CXCR3, physiologically plays a pivotal role in host defense [10,11,12]. Indeed, CXCL10 regulates immune cell activation and recruitment at inflammation sites through CXCR3 subtype A; in human microvascular endothelial cells, which selectively express CXCR3 subtype B, it inhibits angiogenesis [13,14]. With potent chemotaxis, CXCL10 drives the T helper type 1 (Th1)-polarized response initiation and maintenance in autoimmune diseases or in graft injury [15,16]. Its action is directly related to disease pathogenesis and not to a generic inflammatory status [16]. Immune cells represent the main source of CXCL10 but, under inflammatory conditions, it is secreted by several types of tissue cells, like endothelial, keratinocytes, fibroblasts, muscular and cardiac cells, in which normally the chemokine is undetectable [17,18,19,20,21,22,23,24]. CXCL10 production is maximally induced under the synergistic action of tumor necrosis factor (TNF)α and IFNγ [18,19,25,26,27,28].

In the context of rheumatic diseases, CXCL10 exerts pleiotropic functions and promotes multiple injurious processes [29,30].

In fact, in ARD, CXCL10 is known to drive Th1, dendritic cell and monocyte early activity and, in turn, promote detrimental effects, such as joint inflammation, bone destruction, and fibroblast-like synoviocyte invasiveness [15,31,32,33,34].

Patients with RA, SLE, SSc, IIM, Sjogren syndrome (SS), and multiple sclerosis (MS) exhibit higher CXCL10 level in both serum and tissues [17,35,36,37]. Noticeably, CXCL10 may serve as a disease activity marker in early RA and psoriatic arthritis (PsA) and may be an index of injury and severe disease activity in SLE [9,38,39,40,41,42,43]. So far, CXCL10 seems to retain considerable potential as a therapeutic target in the initiation and progression of ARD.

As previously reported in human CD4^+^ T cells, human cardiomyocytes, and human thyrocytes, CXCL10 is modulated by a specific agonist of the transcription factor peroxisome proliferator-activated receptor (PPAR)γ, rosiglitazone (RGZ) [18,25,31,44,45,46].

Although PPARs are primarily recognized for their regulation in lipid and glucose metabolism, increasing attention has been devoted to their role in immune modulation and autoimmune-inflammatory diseases [47,48]. Indeed, these transcription factors, which are sub-grouped in α, γ, and β/δ types, can regulate autoimmune responses by shaping immune cell differentiation and commitment, such as T cell activation, expansion, and differentiation [49].

In particular, PPARβ/δ type is known to act as a “molecular brake” in autoimmune/inflammatory processes; specifically, it can limit pathogenic Th cell dysregulation and expansion, which are acknowledged as key processes contributing to autoimmunity development [49,50,51,52,53]. Activation of PPARβ/δ by specific agonists has been associated with healthy cell development/function and immune-regulatory effects [54].

Nevertheless, differently from PPARα and PPARγ, the potential role of PPARβ/δ activation remains quite unexplored in studies on autoimmunity [49,55].

This study aims to verify whether and how CXCL10 is modulated by PPARβ/δ activation following treatment with the specific agonist carbaprostacyclin (cPGI_2_), a stable analogue of prostacyclin, in human activated CD4^+^ T cells, monocyte-derived dendritic cells (mDCs), and CD14^+^ monocytes, all of which are engaged in autoimmunity development and ARD pathogenesis [56,57,58]. To this purpose, we analyzed CXCL10 protein secretion and gene expression with and without cPGI_2_ in human mDCs, and monocytes, activated with lipopolysaccharide (LPS), and CD4^+^ T cells activated with phorbol 12-myristate 13-acetate (P) and ionomycin (I), respectively.

Under the same experimental condition, IFNγ and TNFα were also assessed, due to their synergistic role in inducing CXCL10 expression. IL-10, a cytokine with regulatory anti-inflammatory effects, was also evaluated [59]. All experiments were performed in parallel with the PPARγ agonist RGZ to verify a possible drug-class-related effect.

## 2. Materials and Methods

### 2.1. Chemicals

RPMI 1640, fetal bovine serum (FBS), phosphate-buffered saline Ca^2+^/Mg^2+^-free (PBS), bovine serum albumin (BSA) fraction V, antibiotics, EDTA trypsin solution, Ficoll-Hypaque, phorbol 12-myristate 13-acetate (P), ionomycin (I), lipopolysaccharide (LPS, E. Coli O111:B4), and reagents for Western blot were from Sigma-Aldrich Corp. (St. Louis, MO, USA). CD4, CD14 isolation kit, and FASER kit PE were from Miltenyi Biotec (Bisley, Germany). PE-anti-CD119 (GIR-208, mouse IgG1) mAb, anti-CD1a, anti-CD3, anti-CD4, anti-CD11c, anti-CD14, anti-CD40, anti-CD80, anti-CD83, anti-CD86, anti–HLA-DR, and isotype-matched control mAbs were from BD Biosciences (Mountain View, CA, USA).

RNeasy MINI reagent kit was from Quiagen Italy (Milan, Italy). TaqMan Reverse Transcription Reagents kit, primer/probe mixes (Taqman Gene^®^ Expression Assays), CXCL10 (Hs00171042-mL), IFNγ (Hs00174143-mL), TNFα (Hs00174128-mL), IL-10 (Hs 00961619-mL), PPAR subtypes α (Hs 00231882-mL), γ (Hs00234592-mL), and βδ (PPAR DELTA: Hs00602622-mL), 1x Universal Master Mix were from Applied Biosystems (Forster City, CA, USA). Quantitative PCR human reference total RNA was from Stratagene (La Jolla, CA, USA). Plasticware for cell cultures were from Corning (Milan, Italy).

### 2.2. Cell Cultures

CD4^+^ T cells and CD14^+^ cells, used to generate mDCs, were obtained and maintained as previously described [60,61,62]. CD14^+^ cells were positively selected from PBMCs by immunomagnetic cell sorting (Miltenyi Biotec, Bisley, Germany). Briefly, mDCs were generated by culturing CD14^+^ monocytes for 7 days in DC-specific media supplemented with 5% FCS (Hyclone, South Logan, UT, USA), 50 ng/mL GM-CSF, and 20 ng/mL IL-4 (R&D Systems, Minneapolis, MN, USA).

These cells were isolated from 5 buffy coats of healthy adult anonymous donors, after Local Ethical Committee approval by Azienda Ospedaliera Universitaria Careggi, Florence, Italy, following the principles outlines in the Declaration of Helsinki. Written informed consent was obtained from all donors. The purity of the sorted population was constantly > 95%.

### 2.3. Protein Secretion Analysis

#### Luminex

mDCs, CD4^+^ T and CD14^+^ cells were seeded and stimulated with LPS [100 ng/mL] (mDCs and CD14^+^ cells) or P/I [1 μM/10 ng/mL] (CD4^+^ T) for 24–48 h, with and without drugs, as previously described [44,60,61]. Cell cultures were exposed to cPGI_2_ [10 μM] or RGZ [5 μM]. After the treatment, cell supernatants were harvested and tested for CXCL10, IFNγ, TNF-α, and IL-10 levels with the Luminex multiplatform bead array (Thermo Fisher Scientific, Austin, TX, USA). Drug concentration was chosen based on the near therapy dose, according to pharmacokinetics (Cmax and area under the time–concentration curve). In each experimental setting, control cells were maintained in the same conditions with vehicles without stimuli.

### 2.4. RNA Extraction and Real-Time Quantitative RT-PCR (TaqMan™)

Cell RNA extraction and analysis were performed as previously described [44]; RNA concentration/quality were evaluated by NanoDrop ND-1000 spectrophotometer (NanoDrop Technologies, Wilmington, DE, USA). TaqMan RT-PCR was performed as described elsewhere [44]. 2^−ΔΔCt^ calculation gave the target amount, normalized to an endogenous reference (GAPDH, pre-developed TaqMan^®^ Assay Reagents) and relative to a calibrator (Quantitavive PCR human reference total RNA) [63].

### 2.5. Statistical Analysis

Statistical analyses were performed using IBM SPSS Statistics version 30.0 (IBM Corp., Armonk, NY, USA). Graphical representations were generated using the latest version of GraphPad Prism 11.1.0 (GraphPad Software, San Diego, CA, USA). Median, interquartile range (IQR), and 25th and 75th percentile (Q1 and Q3) were chosen because of the non-normal distribution of the data. Data normality was assessed using the Shapiro–Wilk test. Non-parametric statistical methods were applied, based on non-normal distribution. Comparisons among groups were performed using the Kruskal–Wallis test, followed by Dunn’s post hoc multiple-comparison test to identify pairwise differences when statistically significant overall effects were observed. A *p* value ≤ 0.05 was considered statistically significant.

## 3. Results

### 3.1. PPARβ/δ Activation Reduced CXCL10 Secretion in Human mDCs and CD4^+^ T

**Figure 1:**
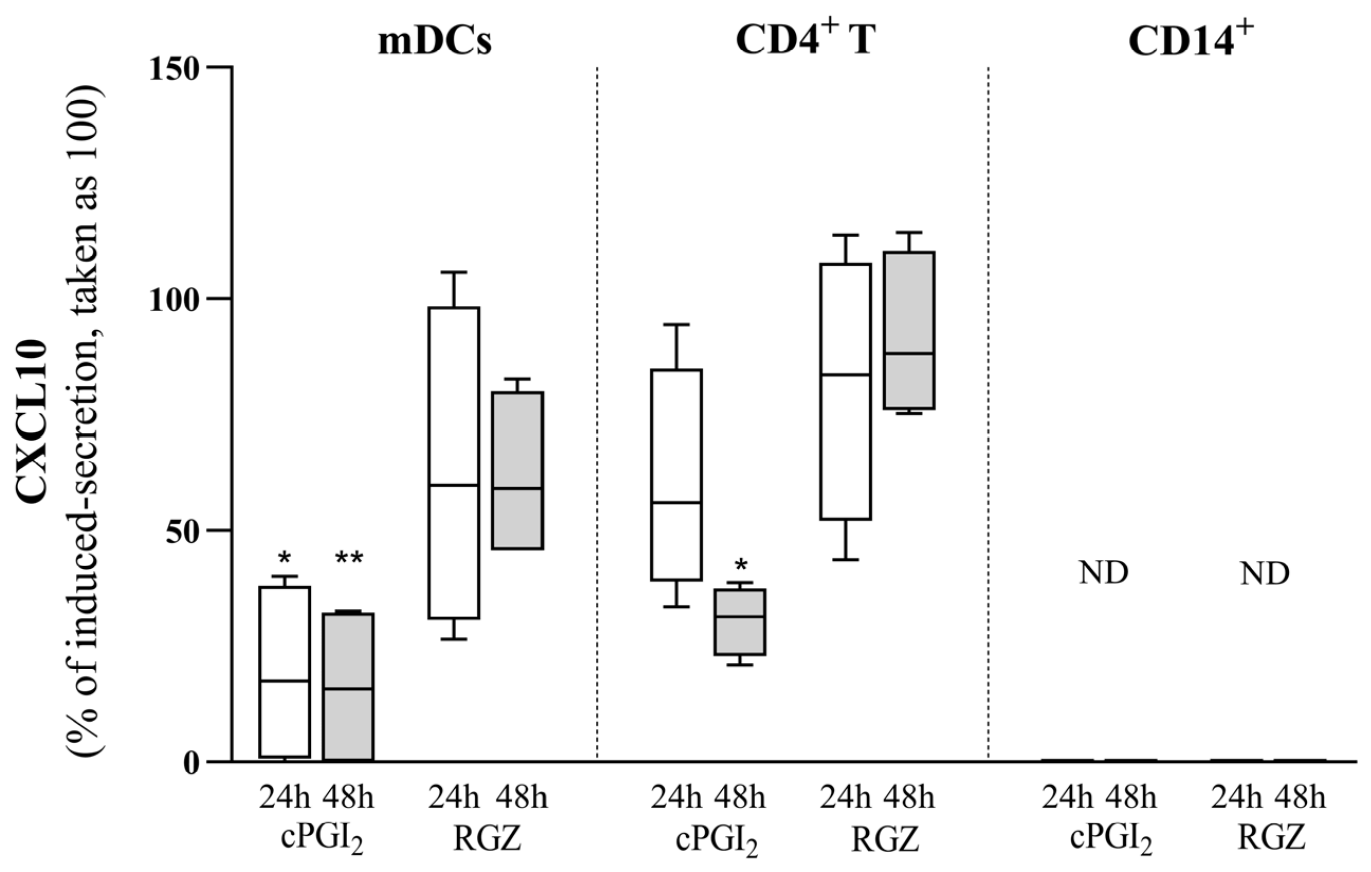
cPGI_2_-induced inhibition of CXCL10 secretion in mDCs and CD4^+^ T cells. CXCL10 release was significantly reduced by cPGI_2_ in mDCs after 24/48 h and in CD4^+^ T cells at 48 h; ** *p* < 0.01 or * *p* < 0.05 vs. CXCL10 induced secretion, taken as 100%. RGZ did not affect CXCL10 protein release tested after 24/48 h in mDCs or CD4^+^ T cells. CXCL10 secretion was not detectable (ND) at each tested time in CD14^+^ cells. Data (N = 4) are expressed as percent of CXCL10 induced secretion, taken as 100%, median (IQR; Q1 and Q3).

Activated mDCs, CD4^+^ T and CD14^+^ were assayed for CXCL10 protein secretion after 24 h and 48 h incubation with cPGI_2_ or RGZ (Figure 1). cPGI_2_ significantly inhibited CXCL10 protein release from mDCs after 24 h, 17.425% (31.96; 2.79 and 34.55%), and after 48 h, 15.770% (31.13; 0.44 and 31.57%) (*p* < 0.05 and *p* < 0.01 vs. induced secretion, taken as 100%). In CD4^+^ T cells, CXCL10 secretion was 56.005% (16.16; 49.90 and 66.07%) after 24 h and 31.280% (8.07; 26.9 and 34.95%) after 48 h (*p* < 0.05 vs. induced secretion, taken as 100%).

RGZ did not affect CXCL10 secretion in mDCs at 24 h, 59.770% (44.27; 39.21 and 83.48%), or 48 h, 59.05% (28.96; 45.86 and 74.82%). Similarly, in CD4^+^ T cells no inhibitory effect was observed with RGZ after 24 h, 83.629% (27.02; 68.88 and 95.90%), or after 48 h, 88.195% (24.77; 77.46 and 102.23%). In CD14^+^, CXCL10 was not detectable (ND) in any tested condition.

Data are derived from four experiments (N = 4) in different cell preparations, run in triplicate and expressed as percent of CXCL10 induced secretion, taken as 100%, median (IQR; Q1 and Q3).

### 3.2. PPARβ/δ Activation Reduced TNFα Secretion in Human DCs, CD4^+^ T and CD14^+^ Cells

**Figure 2:**
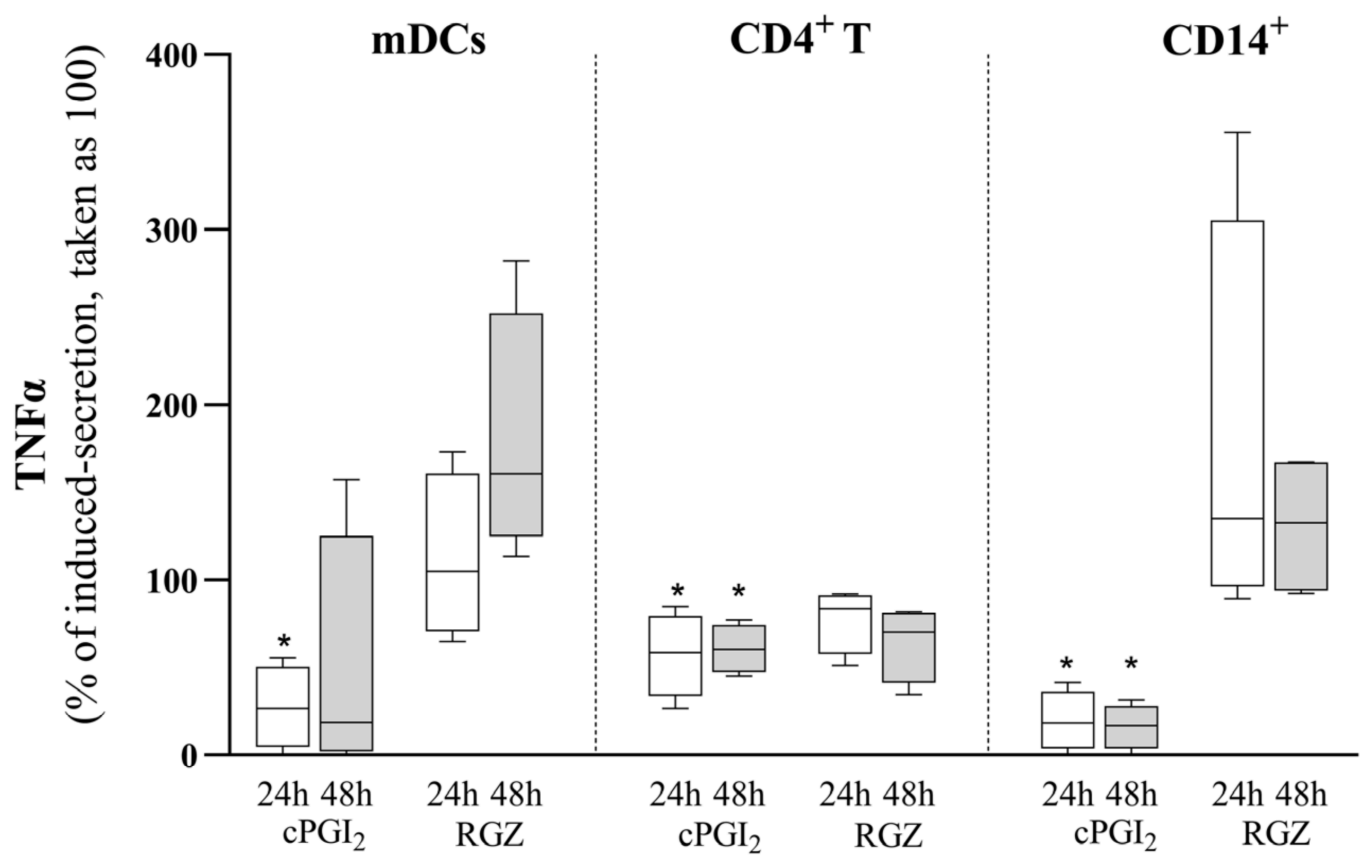
cPGI_2_-induced inhibition of TNFα secretion in mDCs, CD4^+^ T and CD14^+^ cells. TNFα secretion was significantly inhibited by cPGI_2_ in mDCs only at 24 h, whereas in CD4^+^ T at and CD14^+^ cells cytokine release was inhibited at both time points; * *p* < 0.05 vs. TNFα induced secretion, taken as 100%. TNFα protein release did not change in cells exposed for 24/48 h to RGZ. Data (N = 4) are expressed as percent of TNFα induced secretion, taken as 100%, median (IQR; Q1 and Q3).

We tested the effect of PPARβ/δ and PPARγ agonists on TNFα secretion (Figure 2) and found that cPGI_2_ almost halved TNFα release from mDCs after 24 h, 26.860% (45.06; 5.02 and 50.08%) (*p* < 0.05 vs. induced secretion, taken as 100%). Despite the important reduction observed after 48 h, 18.546% (122.76; 2.21 and 124.97%), the statistical significance was not reached for the high IQR.

In CD4^+^ T cells, cPGI2 significantly decreased TNFα release after 24 h, 58.715% (45.05; 34.10 and 79.15%), and 48 h, 60.389% (26.77; 47.47 and 74.24%) (*p* < 0.05 vs. induced secretion, taken as 100%). TNFα protein secretion in CD14^+^ cells exposed to cPGI_2_ was 18.411% (31.96; 4.11 and 36.07%) at 24 h and 16.866% (24.19; 3.97 and 28.16%) at 48 h (*p* < 0.05 vs. induced secretion, taken as 100%). In none of the cell cultures tested RGZ inhibited the induced secretion of this cytokine. Indeed, TNFα release in DCs was 103.33% (87.43; 89.23 and 176.66%) at 24 h and 160.538% (127.05; 124.87 and 251.92%) at 48 h; in CD4^+^ T cells cytokine secretion was 83.730% (33.47; 57.96 and 91.42%) after 24 h and 70.220 (39.67; 41.46 and 81.13%) after 48 h; in CD14^+^ cells protein secretion was 134.850% (208.36; 96.50 and 304.86%) and 132.594% (72.28; 94.41 and 166.70%) after 24 h and 48 h, respectively.

Data are derived from four experiments (N = 4) in different cell preparations, run in triplicate and expressed as percent of TNFα induced secretion, taken as 100%, median (IQR; Q1 and Q3).

### 3.3. PPARβ/δ Activation Reduced IFNγ Secretion in Human mDCs

**Figure 3:**
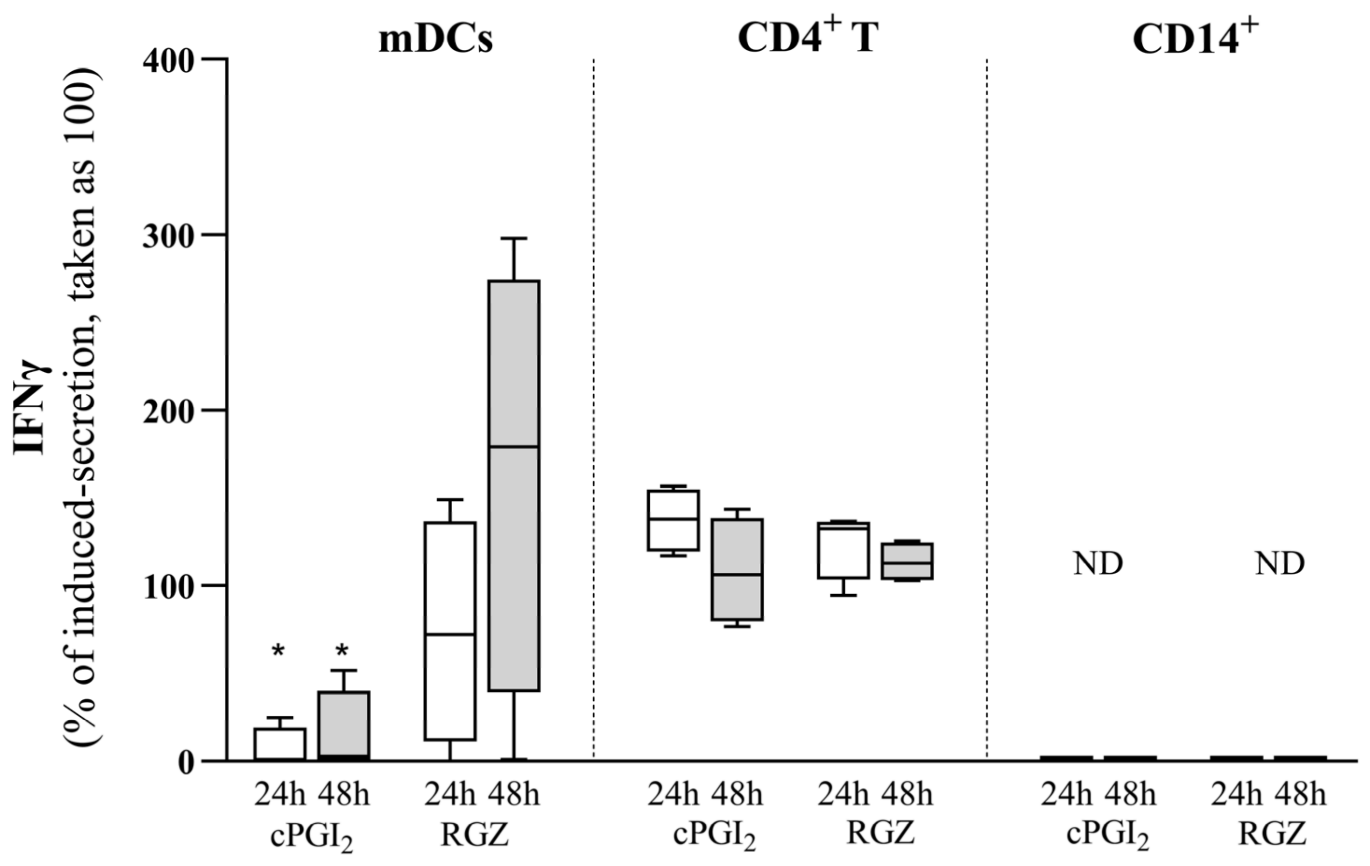
cPGI2 inhibited IFNγ secretion in mDCs. IFNγ release was significantly reduced in mDCs after 24/48 h incubation with cPGI_2_; * *p* < 0.05 vs. IFNγ induced secretion, taken as 100%. Exposure of CD4^+^ T cells to cPGI2 or RGZ for 24/48 h did not modify IFNγ release. In CD14^+^ cells IFNγ was not detectable (ND) at each tested time. Data (N = 4) are expressed as median percent of IFNγ induced secretion, taken as 100% (IQR; Q1 and Q3).

When we evaluated the effect of cPGI_2_ onto IFNγ secretion (Figure 3), in mDCs we observed a significant reduction at both time points, 0.572% (19.095; 0 and 19.095%) at 24 h and 2.936 (39.785; 0.25 and 40.034%) at 48 h (*p* < 0.05 vs. induced secretion, taken as 100%), whereas cytokine release did not change in CD4^+^ T cells after 24 h, 137.884% (35.44; 119.47 and 154.9%), or after 48 h, 106.169 (59.00%; 79.74 and 138.75%). In CD14^+^ cells IFNγ secretion was ND.

In mDCs with RGZ, IFNγ did not change after 24 h, 72.29 (125.25; 11.45 and 137.70%), and showed a trend to increase after 48 h, 179.34 (235.4; 39.3 and 274.7%). In CD4^+^ T cells, RGZ did not modify IFNγ at 24 h, 132.658 (33.00; 103.42 and 136.40%), and at 48 h, 112.873 (21.47; 103.12 and 124.6); IFNγ was ND in CD14^+^ cells.

Data are derived from four experiments (N = 4) in different cell preparations, run in triplicate and expressed as percent of IFNγ induced secretion, taken as 100%, median (IQR; Q1 and Q3).

### 3.4. PPARβ/δ Activation Did Not Change IL-10 Secretion in Human DCs, CD4^+^ T and CD14^+^ Cells

**Figure 4:**
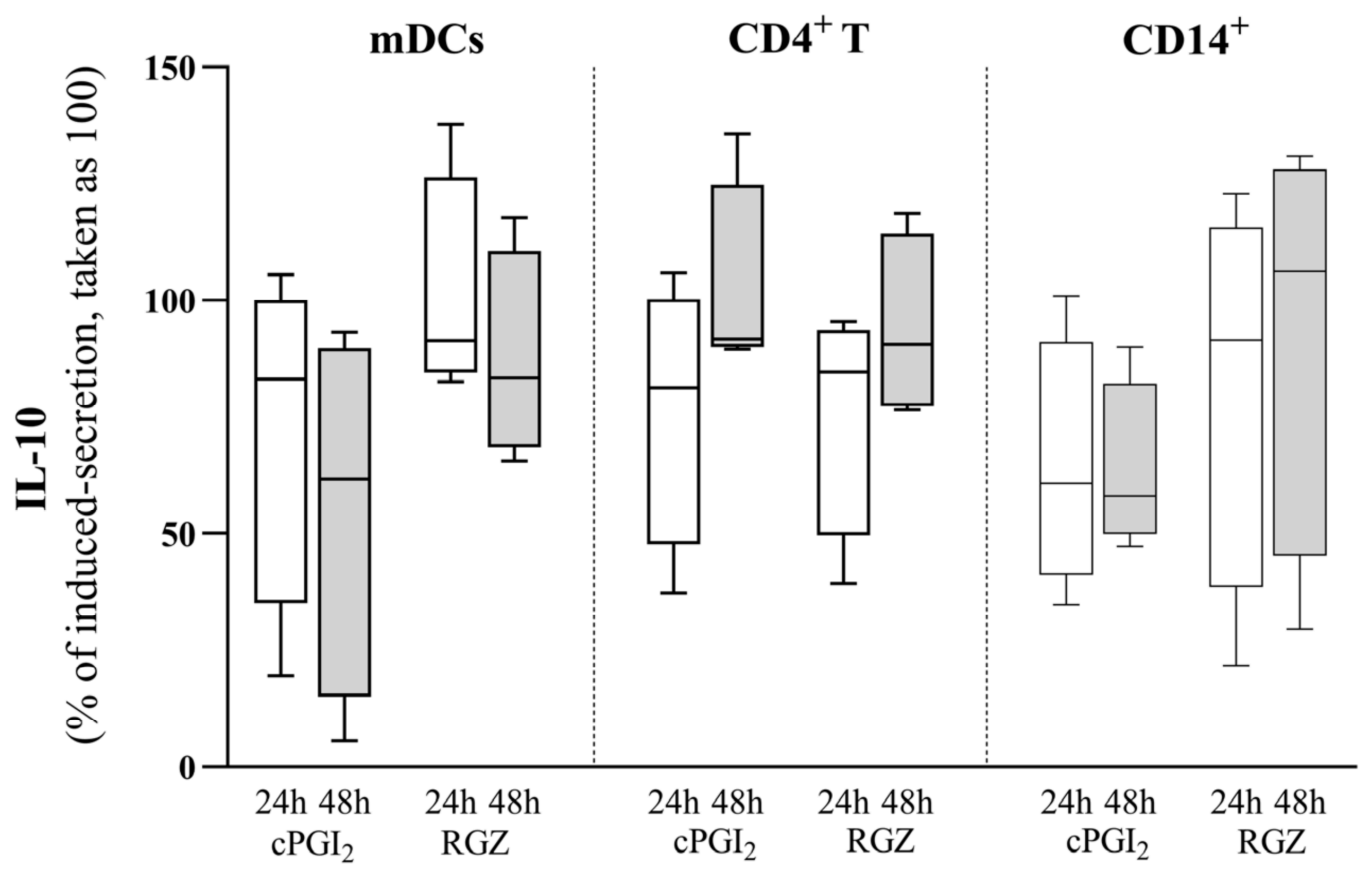
cPGI_2_ did not affect IL-10 secretion in mDCs, CD4^+^ T and CD14^+^ cells. IL-10 protein released amount did not change in activated mDCs, CD4^+^ T and CD14^+^ cells after 24/48 h incubation with cPGI_2_ or RGZ. Data (N = 4) are expressed as percent of IL-10 induced secretion, taken as 100%, median (IQR; Q1 and Q3).

Simultaneously with the proinflammatory cytokines, IL-10 was measured in cell supernatants (Figure 4), since this molecule exhibits anti-inflammatory effects. We observed that IL-10 protein release was not modulated by the presence of both PPARβ/δ and PPARγ agonists. Indeed, in mDCs treated with cPGI_2_ for 24 h and 48 h, IL-10 secreted protein was 83.122% (64.88; 35.20 and 100.08%) and 61.676% (74.74; 15.10 and 89.85%), respectively. Similarly, in CD4^+^ T cells exposed to cPGI_2,_ IL-10 release changed neither after 24 h, 81.255% (52.54; 47.73 and 100.27%), nor after 48 h, 91.800% (34.76; 90.10 and 124.87%). In CD14^+^ cells after cPGI_2_, IL-10 was 60.723% (49.90; 41.17 and 91.08%) at 24 h and 58.085% (32.13; 50.00 and 82.13%) at 48 h.

The treatment with RGZ did not modify IL-10 secretion in mDCs at 24 h, 91.422% (41.92; 84.50 and 126.42%), nor at 48 h, 83.407% (42.00; 68.56 and 110.56%). The drug modified IL-10 secretion neither in CD4^+^ T cells at 24, 84.616% (44.07; 49.60 and 93.68%), and at 48 h, 90.580% (37.04; 77.29 and 114.326%), nor in CD14^+^ cells at both time points: 91.54% (76.98; 38.62 and 115.60%) after 24 h and 106.220% (82.65; 45.36 and 128.01%) after 48 h.

Data are derived from four experiments (N = 4) in different cell preparations, run in triplicate and expressed as percent of IL-10 induced secretion, taken as 100%, median (IQR; Q1 and Q3).

### 3.5. Cytokine mRNA Expression Profile in Human DCs, CD4^+^ T and CD14^+^ Cells After PPARβ/δ Activation

Specific mRNA quantification revealed a significant downregulation of CXCL10, IFNγ and IL-10 expression only in mDCs exposed to cPGI_2_ for 24 h (*p* < 0.05 vs. cytokine induced expression, taken as 1). No other significant changes in cytokine expression were observed in the other experimental conditions (Table 1).

For each cytokine, data derived from four experiments (N = 4) in triplicate were performed in different cell preparations and are expressed as fold increase vs. cytokine induced expression, taken as 1, median (IQR; Q1 and Q3).

### 3.6. Quantification of PPARβ/δ mRNA in mDCs, CD4^+^ T Cells, and CD14^+^ Cells

**Figure 5:**
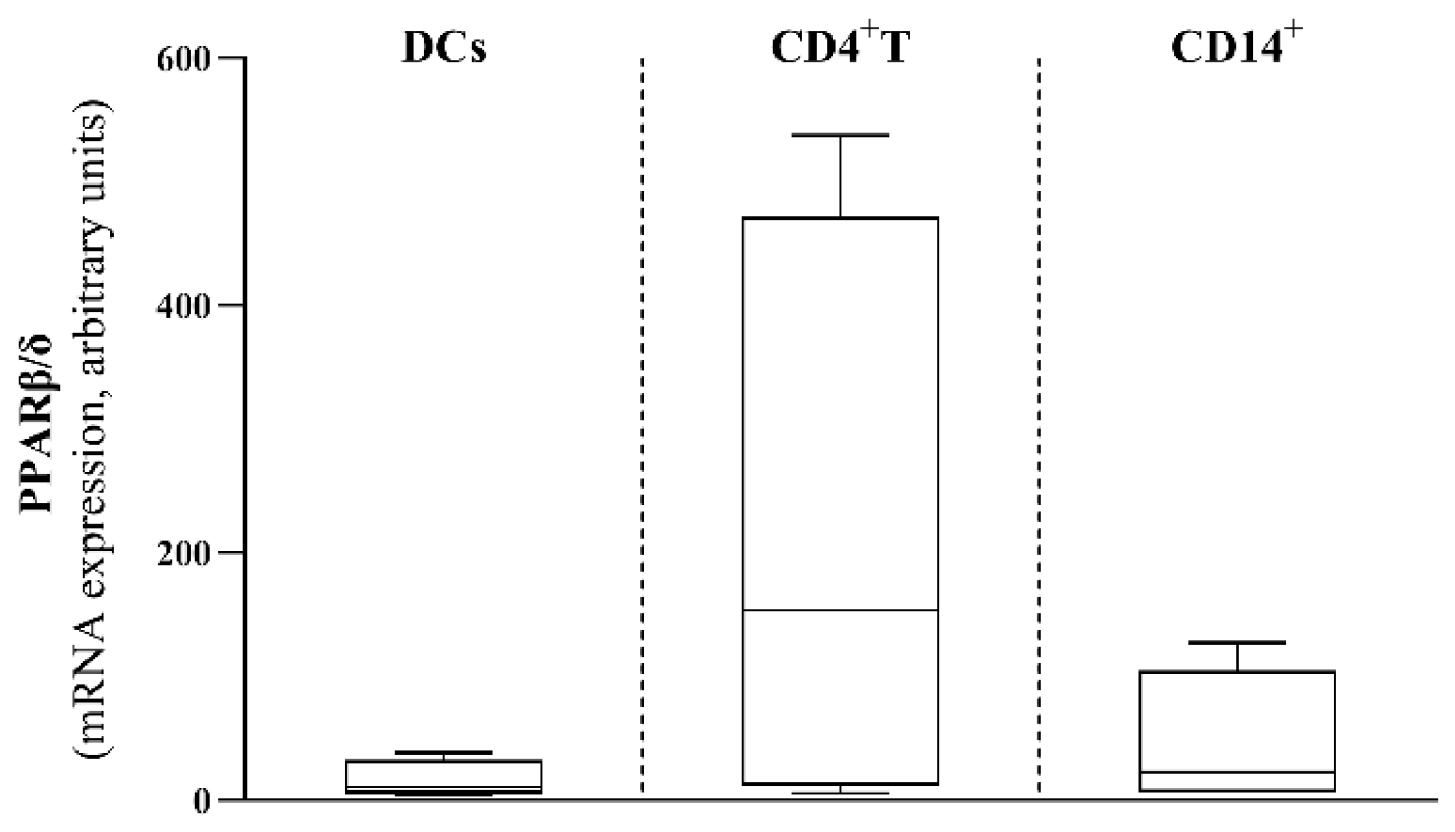
PPARβ/δ expression mRNA in mDCs, CD4^+^ T cells, and CD14^+^ cells. Basal mRNA expression specific for PPARβ/δ in mDCs CD4^+^ T and CD14^+^ cells is depicted. The higher expression is observed in CD4^+^ T cells but not reached statistical significance. Data derived from four experiments (N = 4) in triplicate performed in different cell preparations and are expressed as median (IQR; Q1 and Q3%).

The quantification of basal mRNA specific for PPARβ/δ by qRT-PCR (Figure 5) shows higher expression in human CD4^+^ T cells 153.335% (459.40; 12.05 and 471.45%) vs. mDCs 10.68% (27.54; 4.89 and 32.43%) and CD14^+^ cells 22.51% (98.88; 6.06 and 104.94%), albeit not statistically significant. Data derived from experiments (N = 4) performed in four different cell preparations and are expressed as arbitrary units.

## 4. Discussion

The main finding of this study is that PPARβ/δ selective activation induced by cPGI_2_ translates into a significant CXCL10 protein downregulation in activated human mDCs and CD4^+^ T cells along with a concurrent significant TNFα decrease in mDCs and CD4^+^ T and IFNγ reduction in CD14^+^ cells and mDCs. Conversely, protein secretion of IL-10, a known negative regulator of autoimmune expansion [59], remains unaffected across all evaluated cell types. The data herein reported can contribute to explain the action of PPARβ/δ as a “brake” in autoimmune processes, downregulating CXCL10 both directly and indirectly, through the inhibition of its synergistic inducers TNFα and IFNγ.

Given the early role of CXCL10 in promoting ARD through T cells and DC hyperactivation [15], inhibiting this chemokine as early as possible may be of particular interest. Pre-disease phases of ARD are characterized by dysfunctional DCs, which contribute to the early stages of disease development [64], as well as by T cell dysregulation, resulting in an imbalance between pathogenic effector/memory CD4^+^ T cells and T regulatory cells (Treg) [65,66,67,68,69].

During the early stages of ARD an overproduction of several cytokines/chemokines and soluble mediators occurs, culminating in an aberrant molecule immunoprofiling, commonly referred to as IFN-signature, which is a determinant factor for the autoimmune overresponse [70,71,72]. Once established, this overreactive immunoprofile promotes the breakdown of immunotolerance and subsequent disease development, as observed, i.e., in SLE, SS, IIM, and SSc [73,74,75,76].

To date, the concept of IFN-signature has been revised. Originally, the IFN-signature was referred almost exclusively to type I IFN (mainly IFNα) but, recently, a “mixed” signature, involving type II IFN, particularly IFNγ, has emerged as a prominent feature of the early processes of ARD development and may critically influence the response to pharmacological treatment, as recently reported in SLE, early RA and SSc [73,77,78,79].

In this context, the finding that CXCL10, a main interferon IFNγ-inducible protein, that is, induced by IFNα as well [80,81], is significantly targeted by PPARβ/δ activation claims particular interest since this chemokine may represent an early determinant at the crossroad of IFN-mediated-signaling paths.

CXCL10 can contribute to fine-tuned Th1 cell polarization in autoimmune conditions [82,83]. Increased CXCL10 may represent a “turning point” (cut-off ≥ 165 pg/mL) associated with progression from the preclinical condition, known as very early diagnosis of SSc (VEDOSS), to definite SSc and may also contribute to the development of SSc-related myopathy and cardiomyopathy [17,37]. In subjects affected by SLE, higher serum CXCL10 level correlates with disease activity [84] and is engaged in the pathogenesis of SLE-related organ and tissue manifestations, i.e., renal, cutaneous, and neuropsychiatric ones [85].

In this scenario, the significant inhibition of CXCL10 release induced by cPGI_2_ in human mDCs and CD4^+^ T cells suggests that PPARβ/δ activation may represent a potential strategy to limit the over-reactive cascade.

In addition, the decrease in TNFα observed in human activated dendric cells, lymphocytes and monocytes after cPGI_2_ treatment may be of interest by different aspects, in view of the multifaceted action of this cytokine. Notably, in mDCs exposed to cPGI_2_ for 48 h, TNFα level remained very low after 48 h, although the statistical significance was not reached, likely owing to inter-individual variability and the limited sample size. This issue will likely be overtaken using a larger sample size, as planned in the future experiments.

High levels of TNFα are critical for initiation and maintenance of ARD, as shown in SLE, RA or PsA [86,87]. TNFα dysregulation is associated with deconstruction and damage of organ and tissue architecture, as well as impaired cell apoptosis and clearance of cellular debris, ending in self-antigens and autoantibody formation, as reported in SLE [88].

Indeed, anti-TNFα monoclonal antibodies are included in ARD therapeutic approaches, although their use remains controversial, because of the occurrence of anti-TNFα-induced lupus-like syndrome (ATIL), a severe side-effect paradoxically resembling SLE clinical features [89].

Furthermore, TNFα, through the synergistic action with IFNγ, induces and potentiates CXCL10 release by some organ/tissue resident cells, which normally do not produce this chemokine [17,18,19,20,21,22,23,24]. Local accumulation of CXCL10 boosts, in turn, tissue and organ infiltration via a self-amplifying loop driven by its strong chemoattractant power for CXCR3-positive immunocytes (i.e., T cells, DCs, and natural killer/NK cells). This process is in line with the central role of CXCL10–CXCR3 axis in several inflammatory and autoimmune processes [15,31].

So far, we speculate that cPGI_2_-induced inhibition of TNFα in mDCs, CD4^+^ T and CD14^+^ cells together with the decrease in IFNγ in mDCs could merge in a general down toning of immune overactivation, thereby limiting CXCL10 production both at systemic and local tissue levels.

Targeting DCs may hypothetically have considerable therapeutic potential, given the role of these cells in the early stages of ARD development [15,36,90,91]. DCs dysfunction can lead to the onset of autoimmune diseases, through the expansion of self-reactive effector T cells and follicular Th cells and activation of autoreactive B cells, promoting chronic inflammation and organ end-damage [64]. Thus far, targeting DCs might contribute to control overreactive responses.

These findings are consistent with previous data documenting a general attenuation of immune-inflammatory response through immune cell and cytokine targeting, i.e., suppression of MCP-1 and IL-1β via PPARβ/δ activation promotes macrophage reprograming to a protolerogenic phenotype or DC maturation phenotype with a reduced stimulatory capacity toward T cells [92]. These effects are attributed to immune-modulatory gene regulation exerted through either canonical or inverse regulation (via DNA binding at PPARβ/δ-RXR sites or repression by agonists in the absence of PPARβ/δ DNA binding, respectively) [22,23,24,90,91,92,93,94,95,96].

The decrease in CXCL10 and TNFα secretion in mDCs seems to involve transcriptional regulatory mechanisms, as their specific mRNA expression was downregulated. Post-transcriptional regulation might be involved in protein downregulation of CXCL10 and TNFα in CD4^+^ T and CD14^+^ cells, respectively. Concerning IL-10 mRNA decrease without a corresponding reduction in protein level observed in mDCs and the increase in TNFα mRNA—albeit not significant—found in CD4^+^ T cells, several mechanism(s) might be involved, including alteration in mRNA stability, translational efficiency, transcription rates, differences in mRNA and protein half-lives, and degradation processes.

Further investigations into transcription/processing/storage/degradation, which exert a fine-tuned regulation in protein synthesis rate [97], are warranted. In addition, detailed analyses of the relevant signaling cascade will be necessary to elucidate the mechanism(s) underlying PPARβ/δ agonists’ action.

Moreover, studies into potential cell target-dependent specific effects are mandatory, considering that PPARβ/δ agonists’ activity is highly context-dependent [98,99].

Nevertheless, irrespectively of the underlying mechanisms, the consistent decrease in the secreted protein amount of CXCL10, TNFα and IFNγ following cPGI_2_ treatment might open new perspectives on the possible approach to ARD management. These hypotheses likely warrant further investigations in both clinical and experimental studies.

To date, chemokine signaling is generally regarded as redundant, as one chemokine can interact with multiple receptors and a single receptor can respond to several ligands [100]. Nevertheless, CXCL10 is reported among the relatively few ligands whose neutralization per se can suppress the entire immune/inflammatory process [83,101]. The biological basis of this paradigm remains incompletely understood. The most reliable hypothesis is that individual ligands such as CXCL10 can simultaneously induce different biological actions, including cell mobilization from bone marrow to the bloodstream, selective migration to specific organs, colonization at site of inflammation, and the development or potentiation of specific immune cell populations, including CD4^+^ T cell polarization [95,102]. Hence, multifaceted aspects of autoimmune response would be simultaneously down toned upon the neutralization of this single chemokine. We hypothesize that this process may be particularly relevant in ARD in which CXCL10 is a hub-gene, like in SLE pathogenesis [103].

Concerning PPARβ/δ mRNA expression, the higher level detected in human CD4^+^ T cells does not necessarily translate into a broader biological effect considering at least two relevant issues. First the activity of these transcription factors depends on ligand-induced activation [104]; second, not less important, the magnitude of the biological response is not solely determined by the amount of receptor expressed but depends on the complexity and amplitude of the signaling cascade induced upon ligand–receptor interaction [105].

The inability to detect or quantify CXCL10 and IFNγ secreted protein in CD14^+^ monocytes represents one of the limitations of this study. This cell subtype was mainly used for mDCs generation, and we hypothesize that technical issues may have influenced the analysis of freshly isolated cultures, resulting in an incomplete or potentially biased secretion profile. The lack of comparison with data obtained in cells from patients is another limitation of the study. Herein, we have avoided experiments in cells obtained from ARD subjects to exclude any possible bias due to pharmacological treatments. We plan future studies to reproduce the experiments in a larger sample size including cells isolated from newly diagnosed, treatment-naïve ARD, allowing direct comparison with healthy subjects.

To date, in light of the emerging immunoregulatory effects of PPARβ/δ agonists, the earlier concept of these compounds as “exercise mimetics” with therapeutic potential limited exclusively to metabolic diseases may now be considered outdated [106,107,108]. However, their metabolic effects might be of undeniable interest in ARD, since these diseases are often closely associated with an altered metabolic profile and lipid disorders [109,110]. Therefore, research on PPARβ/δ agonists in the context of ARD appears warranted.

Furthermore, considering that many of the available data derive from studies performed in animal models, which often not adequately recapitulate human disease, further investigations are recommended to address potential species-specific differences and translational biases [111].

## 5. Conclusions

The data reported in this study, although obtained from a relatively small sample size, provide further insight into “molecular brake” action exerted by PPARβ/δ activation. This effect seems to be specific and not drug-class-related, as it was not observed with PPARγ agonist RGZ.

It is mandatory to emphasize that the potential reconsideration of PPARβ/δ agonists for repurposing in the management of autoimmune diseases needs extreme caution, given their pro-tumorigenic properties reported in specific biological contexts. As previously addressed, PPARβ/δ effects are tightly dependent on cellular and molecular environment.

Conclusively, the progress in understanding the biological processes underlying and preceding clinical manifestation in ARD represents a step forward in the challenge to identify future effective therapies to suppress autoreactivity, preventing or even reversing tissue injury caused by the activated immunocytes and their products. Further and more extensive studies into PPARβ/δ agonists, either monotherapy or in combination with other drugs, may open new avenues for exploring their possible utilization in human rheumatic diseases.

## Figures and Tables

**Table 1:** The table summarizes mRNA expression of CXCL10, TNFα, IFNγ, IL-10 in activated mDCs, CD4^+^ T and CD14 ^+^ cells treated with cPGI_2_ or RGZ for 24 h. Data (N = 4) are expressed as fold increase vs. cytokine induced expression, taken as 1, median (IQR; Q1 and Q3). * *p* < 0.05 vs. cytokine induced expression.

mRNA (Fold Increase of Induced Expression, Taken as 1)	mDCs	CD4^+^ T	CD14^+^
**CXCL10**	*cPGI_2_* 0.300 (0.40; 0.04 and 0.44) * RGZ 0.885 (4.78; 0.85 and 5.63)	*cPGI_2_* 0.245 (0.76; 0.09 and 0.85) *RGZ* 0.950 (1.69; 0.16 and 1.85)	*cPGI* 0.140 (0.10; 0.07 and 0.17) *RGZ* 0.520 (1.40; 0.26 and 1.66)
**TNFα**	*cPGI_2_* 0.730 (0.56; 0.26 and 0.80) *RGZ* 1.100 (2.88; 0.33 and 3.22)	*cPGI_2_* 16.275 (38.65; 0.13 and 38.78) *RGZ* 1.140 (5.26; 0.02 and 5.28)	*cPGI_2_* 0.375 (2.44; 0.13 and 2.57) *RGZ* 1.355 (2.06; 0.41 and 2.47)
**IFNγ**	*cPGI_2_* 0.120 (0.21; 0.08 and 0.29) * RGZ 1.010 (0.43; 0.76 and 1.2)	*cPGI_2_* 2.840 (3.15; 1.25 and 4.40) *RGZ* 1.710 (1.59;1.04 and 2.63)	*cPGI* 0.120 (0.4; 0.10 and 0.50) *RGZ* 2.200 (3.23; 1.34 and 4.57)
**IL-10**	*cPGI_2_* 0.210 (0.16; 0.15 and 0.30) * RGZ 0.920 (0.11; 0.86 and 0.97)	*cPGI_2_* 2.600 (0.95; 1.95 and 2.90) *RGZ* 1.215 (3.57; 1.06 and 4.63)	*cPGI_2_* 0.590 (0.72; 0.28 and 1.00) *RGZ* 1.105 (0.46; 0.83 and 1.29)

## Data Availability

The original contributions presented in this study are included in the article. Further inquiries can be directed to the corresponding author.

## Abbreviations

The following abbreviations are used in this manuscript:

ARD	Autoimmune rheumatic diseases
ATIL	Anti-TNFα-induced lupus-like syndrome
BSA	Bovine serum albumin
cPGI2	Carbaprostacyclin
CXCL10	C-X-C motif chemokine ligand 10
CXCR3	C-X-C motif chemokine receptor 3
DCs	Dendritic cells
mDCs	monocyte-derived dendritic cells
EDTA	Ethylenediaminetetraacetic acid
ERL	Glutamic acid–leucine–arginine
FBS	Fetal bovine serum
GAPDH	Glyceraldehyde-3-phosphate dehydrogenase
HLA-DR	Human leukocyte antigen-DR
IFN	Interferon
IFNγ	Interferon gamma
IIM	Idiopathic inflammatory myopathies
IL-10	Interleukin-10
IP-10	IFNγ-induced protein 10
IQR	Interquartile range
LPS	Lipopolysaccharide
mAb	Monoclonal antibody
MCP-1	Monocyte chemoattractant protein-1
mRNA	Messenger RNA
MS	Multiple sclerosis
ND	Not detectable
NF-κB	Nuclear factor kappa B
PBS	Phosphate-buffered saline
PCR	Polymerase chain reaction
P/I	Phorbol 12-myristate 13-acetate/Ionomycin
PPARα	Peroxisome proliferator-activated receptor alpha
PPARβ/δ	Peroxisome proliferator-activated receptor beta/delta
PPARγ	Peroxisome proliferator-activated receptor gamma
PsA	Psoriatic arthritis
qRT-PCR	Quantitative reverse transcription polymerase chain reaction
RA	Rheumatoid arthritis
RGZ	Rosiglitazone
RNA	Ribonucleic acid
SLE	Systemic lupus erythematosus
SSc	Systemic sclerosis
SS	Sjögren syndrome
STAT1	Signal transducer and activator of transcription 1
Th1	T helper type 1
Th17	T helper type 17
TNFα	Tumor necrosis factor alpha
Treg	Regulatory T cell
VEDOSS	Very Early Diagnosis of Systemic Sclerosis
